# Autoimmune Channelopathies at Neuromuscular Junction

**DOI:** 10.3389/fneur.2019.00516

**Published:** 2019-05-17

**Authors:** Kun Huang, Yue-Bei Luo, Huan Yang

**Affiliations:** ^1^Neurology Department, Xiangya Hospital, Central South University, Changsha, China; ^2^Division of Neurogenetics, Center for Neurological Diseases and Cancer, Nagoya University Graduate School of Medicine, Nagoya, Japan

**Keywords:** neuromuscular junction (NMJ), channelopathies, Lambert-Eaton syndrome (LEMS), Isaacs' syndrome, myasthenia gravis (MG)

## Abstract

The neuromuscular junction, also called myoneural junction, is a site of chemical communication between a nerve fiber and a muscle cell. There are many types of channels at neuromuscular junction that play indispensable roles in neuromuscular signal transmission, such as voltage-gated calcium channels and voltage-gated potassium channels on presynaptic membrane, and acetylcholine receptors on post-synaptic membrane. Over the last two decades, our understanding of the role that autoantibodies play in neuromuscular junction disorders has been greatly improved. Antibodies against these channels cause a heterogeneous group of diseases, such as Lambert-Eaton syndrome, Isaacs' syndrome and myasthenia gravis. Lambert-Eaton syndrome is characterized by late onset of fatigue, skeletal muscle weakness, and autonomic symptoms. Patients with Isaacs' syndrome demonstrate muscle cramps and fasciculation. Myasthenia gravis is the most common autoimmune neuromuscular junction channelopathy characterized by fluctuation of muscle weakness. All these disorders have a high risk of tumor. Although these channelopathies share some common features, they differ for clinical features, antibodies profile, neurophysiological features, and treatments. The purpose of this review is to give a comprehensive insight on recent advances in autoimmune channelopathies at the neuromuscular junction.

## Introduction

Neuromuscular junction (NMJ) is a type of chemical synapse between motor neurons and skeletal muscles, which consists of presynaptic membrane, synaptic cleft, and post-synaptic membrane. The most crucial event at NMJ is neuromuscular transmission that leads to contraction of skeletal muscles. In order to contract skeletal muscles, chemical neurotransmitters, such as acetylcholine (ACh), are released from presynaptic membrane, under the synergy of ion channels, such as voltage-gated calcium channels (VGCCs) and voltage-gated potassium channels (VGKCs), to post-synaptic membrane, binding to acetylcholine receptors (AChRs) of which the clustering and maintenance need muscle-specific kinase (MuSK), lipoprotein-related protein 4 (LRP4), and agrin (1). Neuromuscular junction channelopathies include a variety of disorders of genetic, toxic, and autoimmune origin. Regardless of the causes, these disorders lead to an impaired neuromuscular transmission. Acquired autoimmune channelopathies at neuromuscular junction include Lambert-Eaton syndrome (LEMS), Isaacs' syndrome, and myasthenia gravis (MG).

LEMS is caused by an autoimmune attack against presynaptic VGCCs and is characterized by late onset of fatigue, skeletal muscle weakness, weight loss, autonomic dysfunction, and areflexia. It develops in the context of a malignant neoplasm, usually small cell lung carcinoma (SCLC) (2). Isaacs' syndrome is caused by autoantibodies against VGKCs and patients with Isaacs' syndrome complain of muscle stiffness and cramps, and on physical examination demonstrate fasciculation (3). MG is an autoimmune disease associated with antibodies usually directed against AChRs, MuSK, or LRP4, in the post-synaptic membrane at NMJ, and is characterized by fluctuation of muscle weakness and fatigue (4).

Except for Isaacs' syndrome, although these channelopathies share some symtoms, such as skeletal muscle weakness and fatigue, they differ for clinical features, antibodies profile, neurophysiological features, and treatments. In this paper, we mainly focus on the clinical, laboratory, and pathological features, as well as treatment of these channelopathies, and give a comprehensive insight on recent advances in autoimmune neuromuscular junction channelopathies.

## NMJ

### Structure and Function of the NMJ

**Figure 1:**
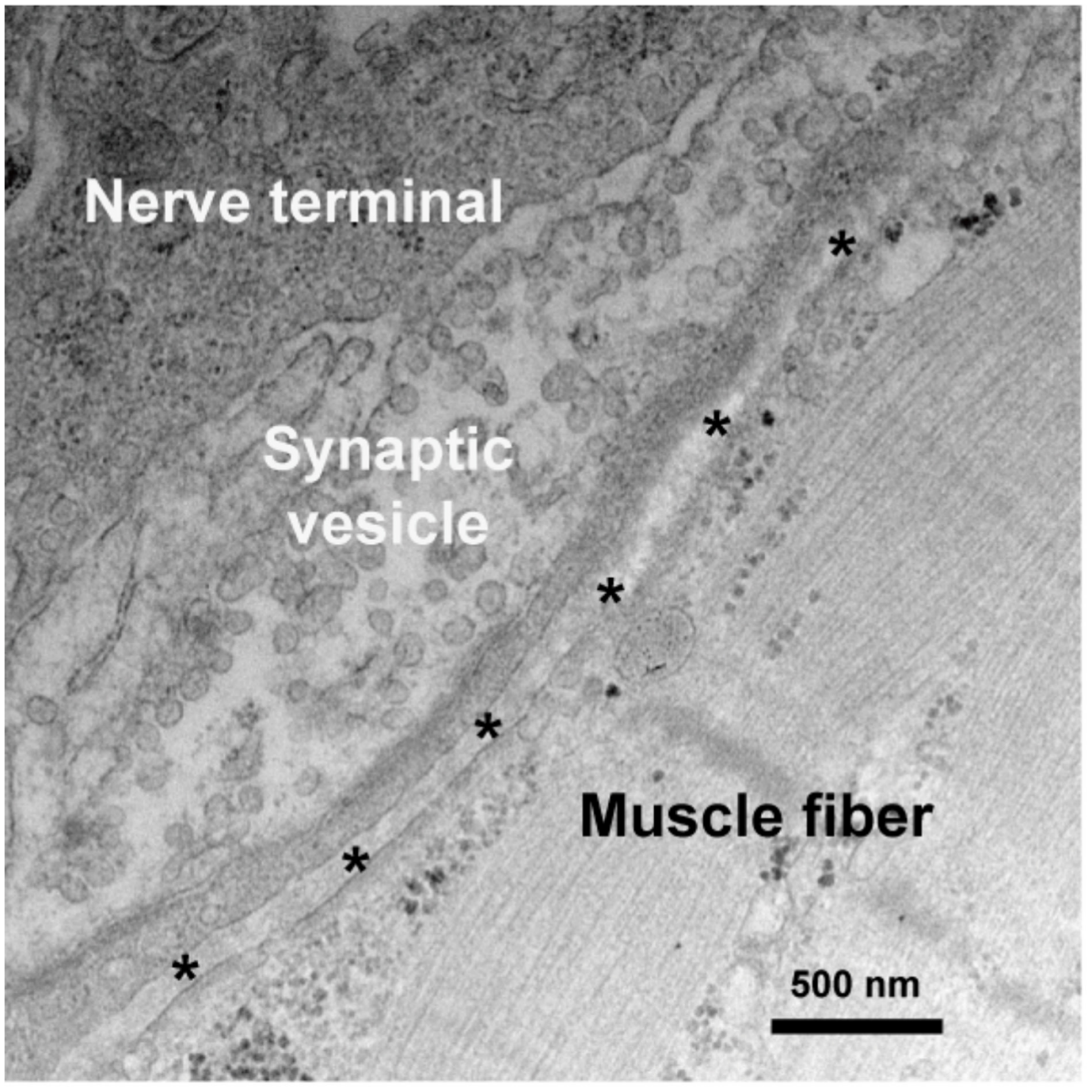
Synaptic ultrastructure at the NMJ. A representative electron micrograph of a human NMJ synapse. Asterisks represent the synaptic cleft.

The NMJ, also called myoneural junction, is a specific chemical synapse site between nerve terminal and muscle fiber, causing muscle contraction through transmitting signal from the motor neuron to muscle fiber (5). NMJ, which typically locates near the middle of the muscle fiber, consists of three parts, presynaptic membrane, synaptic cleft, and post-synaptic membrane (Figure 1).

#### Presynaptic Membrane Channels

##### VGCCs

**Figure 2:**
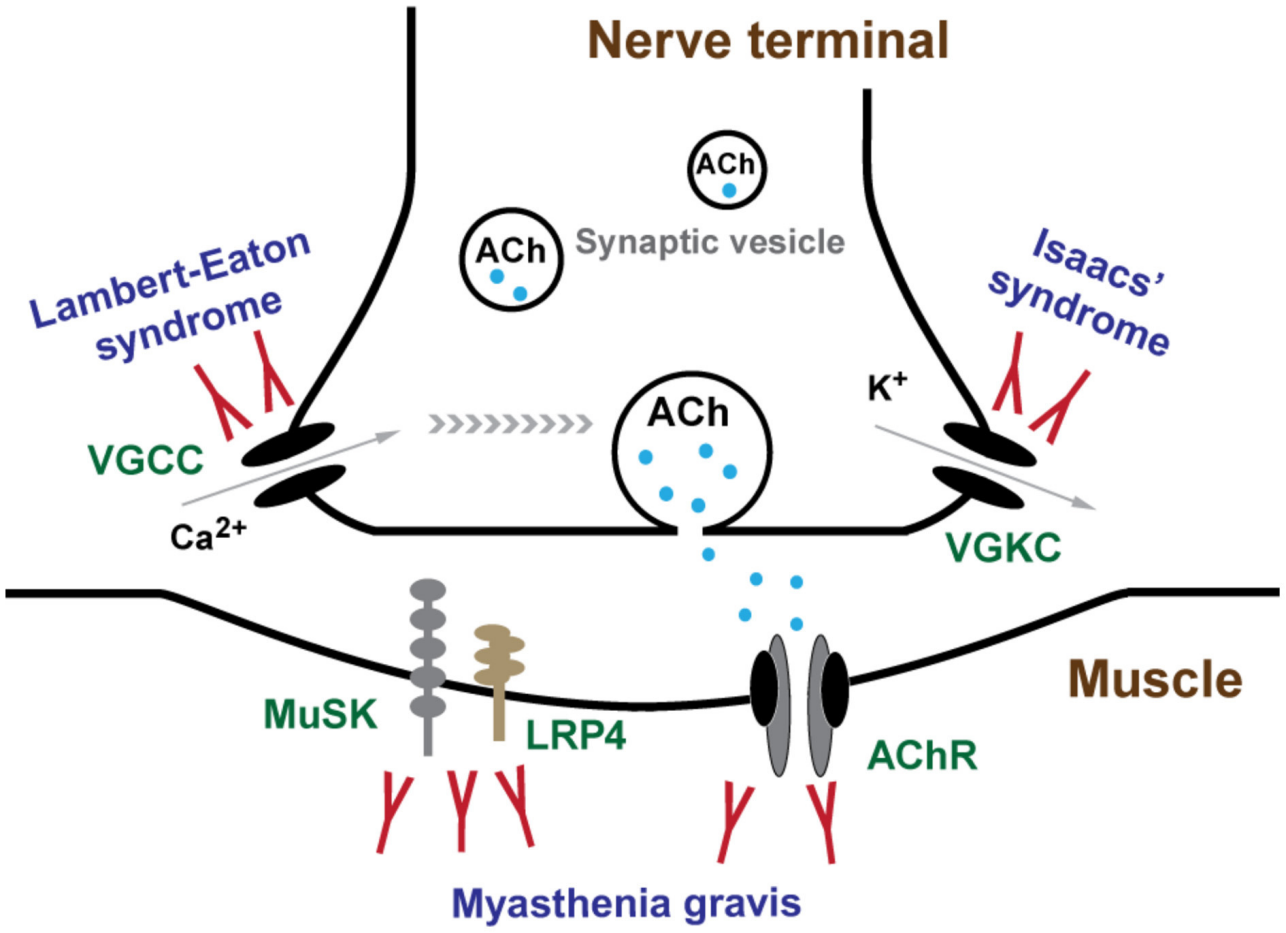
Channels and channelopathies at NMJ. NMJ channels and their associated human diseases are indicated in green and blue letters, respectively. The entry of calcium ions (Ca^2+^) through VGCCs depolarize the nerve terminal, resulting in the release of ACh from synaptic vesicles into synaptic cleft. In repolarization process, potassium ions (K^+^) leak out to the extracellular through VGKCs, accompanied by a recovery of resting potential and a halt of substantial ACh release. Diffused ACh are captured by AChR leading to muscle contraction. LEMS and Isaacs' syndrome are caused by antibodies against VGCCs and VGKCs, respectively. MG is mainly caused by antibodies against AChR, MuSK, and LRP4.

VGCCs is a group of voltage-gated ion channels with a preferential permeability to the calcium ions and are also slightly permeable to sodium ions (Figure 2) (6). One of the essential factors underlying neurotransmitter release and nerve conduction at the presynaptic membrane is the calcium dynamics. VGCC is a complex protein consisting of multiple subunits. The pore-forming α1 subunit is responsible for the biochemical and electrophysiological characteristics of VGCC. At physiological or resting membrane potential, VGCCs are normally closed, the concentration of calcium ions is much lower in inside of the presynaptic membrane than outside (7). During an action potential, VGCCs are activated and open, causing a substantial and temporary influx of the calcium ions and a surge of calcium concentration, then calcium ions flow away from the channel and interact with neurotransmitter release sensors, calcium buffering proteins or kinases (8, 9).

##### VGKCs

VGKCs are transmembrane channels sensitive to voltage changes and specific for potassium in membrane potential (Figure 2). Each subunit of VGKCs has six transmembrane regions named S1 to S6, with N- and C- terminals located at the intracellular side. The channel pore is surrounded by the S5 and S6 regions. Between the region S5 and S6, there is a P region which associates with S6 to form a selectivity filter of the ions (10). VGKCs are involved in determination of the resting potential of cell membranes, controlling thresholds of excitation, modulating wave forms and frequencies of action potentials, and repolarization of depolarized membranes (11). The resting membrane potential of mammalian nerve terminal is generally close to the potassium equilibrium potential owing to the function of background VGKCs. During action potentials, VGKCs play an important role in returning the depolarized cell to a resting state by removing the redundant potassium outside (12). Antibodies of VGKCs in peripheral nervous system cause autoimmune neuromyotonia disorders, such as Isaacs' syndrome, and in central nervous system lead to Morvan syndrome (13) and limbic encephalitis (14).

#### Post-synaptic Membrane Channels

##### AChRs

Post-synaptic membrane, namely the sarcolemma, harbors a high density of transmitter receptors, such as AChRs in a density of almost 10,000 AChRs/μm^2^ (15). Clustering of AChRs at the NMJ is mediated by the agrin-LRP4-MuSK signaling (5, 16). LRP4 is a member of the LDL receptor family. Neural agrin does not directly bind to MuSK, but it activates MuSK through binding with LRP4 (17). MuSK binds to LRP4 to receive neural agrin signaling which is essential for clustering and function maintenance of AChR. AChRs are distributed spatially restricted to the area immediately surrounding the opening of post-junction folds and partially down the sides of the infolded membrane and excluded from the trough of post-junctional folds (18).

There are mainly two types of AChRs, nicotinic acetylcholine receptors (nAChRs) and muscarinic acetylcholine receptors (mAChRs) in human. nAChRs are ion channels which allow the trafficking of sodium, potassium and calcium ions with no selectivity for cations, while the mAChRs are not ion channels (19). The nAChRs are pentamers containing four macromolecules, such as cationic AChRs, cationic serotonergic receptors (5HT3), anionic glycine receptors, and anionic GABA_A_ and GABA_C_ receptors (20). Subunits of nAChRs in neurons and muscles are different. Muscular nAChRs are comprised of, two α subunits, and one each of β, γ and δ in the fetal type, resulting in a stoichiometry of α_2_β*δγ*, while in the adult type, the ε subunit replaces the γ subunit with a stoichiometry of α_2_β*δε* (21). The relative contents of the two types of AChR channels, depend on innervation of the muscle by spinal motor neurons. Before innervation, the fetal type predominates; at later stages of synapse formation, the fetal type is replaced by the adult type (22, 23). The adult type appears during the first post-natal week and replaces majority of the neonatal form within the second post-natal weeks except for a small part skeletal muscle, such as some extraocular muscles (24). During the time of transition, endplates have both neonatal and adult types of AChR (25, 26). Genes code for these subunits include: *CHRNA1* for α, *CHRNB* for β, *CHRND* for δ, *CHRNG* for γ, and *CHRNE* for ε. Antibodies of AChRs usually lead to MG.

### Nerve Impulse Conduction at NMJ

The entry of calcium ions through VGCCs serves as a connection between the depolarization of the nerve terminal and the activation of the neurotransmitter release mechanism (27). A single nerve impulse conducts to nerve terminal, immediately causes the activation of VGCCs which are responsible for calcium action potentials, with an influx of calcium into the intracellular side. Calcium influx causes vesicular exocytosis, leading to ACh release from the vesicles to synaptic cleft (28, 29). The ACh release process triggered by the calcium ion influx is mediated by 100–300 synaptic vesicles, and raises the local concentration of ACh in the synaptic cleft to a concentration of almost 0.3 mM (30). In repolarization process, an important event is potassium ions leak to the extracellular side as a result of activation of VGKCs, accompanied by a recovery of resting potential and a halt of substantial ACh release. AChs, the 146Da small molecules released from the nerve terminal in bursts, diffuse immediately into the synaptic cleft and are captured by AChRs, binding to the α subunits of AChR at their interfaces with surrounding γ and δ subunits (31). Then AChRs are activated and open in microseconds with a flux of cations, mainly sodiums, flowing through by their electrochemical gradients (32). This causes a depolarization potential, which induces an action potential and contraction in the muscle fiber it controls. Normally, a myriad excess of ACh is released from the presynaptic membrane, and several times as many AChRs are activated as would be necessary for an endplate potential (EPP) to reach the muscle-fiber firing threshold. The redundant AChs in the synaptic cleft are hydrolyzed by acetylcholinesterase (AChE) within a millisecond. The opening and closing of AChRs are only too quick to result a prompt initiation and termination of the post-synaptic response (33). Nerve impulse conduction at NMJ are indicated in Figure 2.

Safety margin of neuromuscular transmission is generally defined as the ability of neuromuscular transmission to remain effective under various physiological conditions and stresses (34). In the case of NMJ, a large surplus of both ACh and AChR provides a safety margin which allows threshold depolarization across every stimulated NMJ under normal circumstances (35). Once the excess AChRs have been blocked leading to a decreased safety margin, the released ACh cannot produce a sufficient strong signal to generate a signal in the post-synaptic membrane to cause a muscle contraction (36). Autoimmune or genetic defects at the presynaptic region, synaptic basal lamina, or post-synaptic structure of the neuromuscular junction can impair the safety margin of neuromuscular transmission. In MG, antibodies against AChRs lead to a decreased safety margin of neuromuscular transmission, so that slight depletion of ACh results in failure of post-synaptic depolarization for many muscle fibers. Similarly, in LEMS, antibodies against VGCC compromise the safety margin which results in radically decrease ACh release at all times. By comparison, in Isaacs' syndrome, antibodies against VGKC lead to delayed repolarization of the axon after each action potential impairing safety margin which contributes to prolongation of the depolarization of the muscle fiber membrane (34).

Since different channels play varying roles at NMJ, autoimmune antibodies of certain channels cause distinct symptoms. Antibodies against VGCCs and AChRs usually cause similar symptoms, such as skeletal muscle weakness, to list the main feature, due to insufficient AChs released from presynaptic membrane or reduced functional AChR density on post-synaptic membrane, respectively (37). While antibodies against VGKCs often lead to serial symptoms as a result of redundant AChs released from the nerve terminal, such as muscle stiffness and cramps (3).

## LEMS

LEMS is an autoimmune neuromuscular junction channelopathy caused by antibodies against VGCCs. Symptoms mainly include late onset muscle fatigue and weakness, weight loss, and autonomic symptoms, such as dry mouth, male impotence, and constipation, usually in association with malignant tumor (38). This rare channelopathy was first reported by Edward Lambert and Lee Eaton in 1957, with a distinctive electrophysiological abnormalities in repetitive nerve stimulation (RNS) which were remarkably different from that of typical MG (39). Approximately 50% of LEMS patients have a primary autoimmune disorder and 60% of patients with LEMS have a tumor, most often SCLC (40). Since almost half of the LEMS associated with tumor, LEMS was usually categorized as non-tumor LEMS (NT-LEMS) and paraneoplastic LEMS (CA-LEMS). Some LEMS clinical symptoms overlap with those of other myasthenic syndromes, most commonly MG, which may lead to misdiagnosis or delayed diagnosis.

### Epidemiology

Since LEMS is a rare channelopathy, the epidemiological data varies with different district, usually with a world-wide prevalence of 2–4 per million, which is ~46 times less than that of MG (41–43). The median age of onset is around 50–60, but LEMS can also affect children (44, 45). Particularly, a female predominance has been found in individuals diagnosed under 45 years. On the contrary, a male predominance in those diagnosed after the age of 60 years (46). In CA-LEMS, the median age of onset is 60 years with a male predominance, while in NT-LEMS, the first peak age of onset is around 35 years old and a second, larger peak is age 60 years. The age and sex distribution in NT-LEMS is similar to that reported for MG (47).

### Clinical Features

The clinical triad of LEMS typically consists of proximal muscle weakness, autonomic features, and areflexia (40). Patients with LEMS almost invariably suffer from proximal weakness of lower limbs as a first symptom. Gradually, upper limbs, distal lower limbs and sometimes cranial muscles are also involved. As a hallmark of MG, ptosis can also be detected in LEMS, albeit generally in a mild form and later in the disease course (48). Since the main clinical features are similar with those of subacute myopathy, also electromyography (EMG) and biopsy abnormalities mimicking myopathy may often be found in patients with LEMS, therefore it is of obvious importance to diagnose LEMS patients from myopathy (49). The disease progression is much more malignant in CA-LEMS than in NT-LEMS. Usually legs and arms are implicated since the onset of symptoms in a large percentage of CA-LEMS, while most of NT-LEMS may only have proximal lower limbs weakness. Although artificial ventilation was reported as approximately in 11% of LEMS, respiratory failure, a common manifestation of MG, is infrequent in LEMS and it is always due to paralytic agents, such as pancuronium, atracurium, and vecuronium, use or intercurrent pulmonary pathology (50, 51).

Autonomic dysfunction is reported in up to 96% of patients with LEMS (40, 44, 47, 52, 53). Dry mouth, constipation, and erectile dysfunction in men are particularly common, and loss of sweating, orthostatic hypotension, and pupillary abnormalities can also be found. Another typical symptom is the decreased or absent tendon reflexes in LEMS. Deep tendon reflexes are always reduced or absent, especially in the lower limbs. In up to 40% of patients with LEMS, a previously absent or significantly reduced deep tendon reflex will return to normal, also with a recovery of muscle strength to almost normal level, after 10 s of maximal voluntary contraction, which is a characteristic phenomenon in LEMS (38). Thus, tendon reflexes should be tested after a period of rest because of the post-exercise facilitation phenomenon can disguise the abnormal tendon reflexes.

### Pathophysiology

LEMS consists of NT-LEMS and CA-LEMS. Tumor association is estimated in about 60% of patients with LEMS (51). The most common malignant carcinoma of CA-LEMS is SCLC, a smoking-related neuroendocrine lung carcinoma. Other tumors also have been found associated with LEMS, such as non-small cell and mixed lung carcinomas, prostate carcinoma, and thymoma (40, 46, 51). Since these cancers have neuroendocrine characteristic, antibodies against VGCC subunits were generated during the disease duration. Besides, SOX1 protein plays a role in airway epithelial differentiation and is shown to be present in SCLC, which show a relative good value for LEMS diagnosis.

#### Antibodies

Until 1983, the pathogenic antibodies against VGCCs was first found by Fukunaga (54). The discovery of pathogenic autoantibodies of VGCC has greatly facilitated diagnosis of LEMS and improved the understanding of the underlying pathophysiologic mechanisms. Subsequent researches show the most popular antibodies are that against P/Q-type VGCCs, which cause most of the clinical symptoms of LEMS (55). However, the significance of an elevated antibodies against VGCC titer beyond its original clinicopathological correlate, LEMS remains undetermined.

Traditionally, antibodies against P/Q-type VGCCs are detected in 85–90% of patients with LEMS, in some reports even with up to 100% in LEMS patients with SCLC, which suggests a high specificity of LEMS diagnosis (55–57). Interestingly, recent researches reported that antibodies against VGCC were detected not only in other autoimmune diseases, such as MG, but also in healthy people, which questioned the specificity of antibodies against VGCC for LEMS diagnosis. Di Lorenzo found that antibodies against P/Q-type VGCC had a diagnostic sensitivity of 88.89% and specificity of 36.17% (58). Zalewski also reported that antibodies against P/Q-type VGCC have a compromised specificity on LEMS diagnosis (59). Antibodies against another type of VGCCs, N-type or L-type VGCCs, have also been found in 30–40 and 25% of LEMS patients, respectively, but all of these patients were also be detected the P/Q-type VGCCs antibodies (57, 60). Although antibodies against P/Q-type VGCCs are somehow highly sensitive to LEMS, since it has a low specificity, cautious interpretation of results, particularly medium and low titers, is advised.

SCLC itself expresses three types of VGCCs, the N, L, and P type (61). Because SCLC is of neuroendocrine origin, it expresses the same types of VGCCs and secretory machinery as nerve terminals. Immune system produces antibodies targeting the protein which are secreted by SCLC, also attacking VGCCs on motor nerve terminal. The P/Q type of VGCCs, and also N type of VGCCs, are two main targets of IgG-mediated nerve terminal autoimmunity in LEMS (59).

In recent years, a new marker, SOX1, associated with paraneoplastic neurological disease has been described (62, 63). SOX1 is thought to prevent neural differentiation in progenitor cells and mainly expressed in the developing nervous system and downregulated in adults (64). Two studies showed antibody against SOX1 presents in 64–67% of patients with SCLC-LEMS, compared to 0–5% in NT-LEMS patients (65, 66). Using ELISA assay, SOX1 antibody has a sensitivity of 67% and a specificity of 95% to discriminate between SCLC-LEMS and NT-LEMS (66).

A small part of LEMS patients have no detectable VGCCs antibodies, namely the seronegative LEMS. Although antibodies were undetectable in seronegative LEMS, the clinical phenotype is almost identical to seropositive LEMS patients (67). Since passive transfer of seronegative LEMS sera to mice can also generate the typical symptoms and electrophysiological changes as those passively transferred with seropositive sera, seronegative LEMS might therefore due to the same antibodies of VGCCs but at a relatively lower titer, or other antibodies of VGCCs' epitopes not recognized currently (38). Intriguingly, antibodies against AChRs can also be detected in a small part of LEMS, while these specific antibodies have no diagnostic value (68).

## EMG

Needle electrode EMG examinations are necessary for patients suspected of having disorders of synaptic transmission, such as LEMS, MG, and Isaacs' syndrome. RNS is essential for the diagnose of LEMS. In LEMS, the first compound muscle action potential (CMAP) is low, even lower at stimulating frequencies, about 2–5 Hz (69). Mostly, reduction of CMAP amplitude of 10% is considered abnormal. In LEMS, almost all the patients show a massive decrease of CAMP (38, 68). One of the key method to differentiate LEMS and MG is the high-frequency stimulation (50 Hz). An increase of the CMAP amplitude more than 100% is considered specific for LEMS. More recently, it is suggested that the threshold for LEMS diagnose can be decreased to 60% to improve sensitivity to 97% while retaining specificity of 99% to exclude MG (70). Single-fiber electromyography (SFEMG) is slightly more sensitive than RNS for diagnosis of LEMS (70). However, SFEMG is less specific than RNS and requires technical experience (69).

### Treatment

Most of the LEMS patients have concomitant cancers, so treatment should include two parts, treatment against the known tumor when applicable and symptomatic management.

#### Oncological Screening

More than half of LEMS patients are associated with SCLC, thus it is pivotal to screen underlying tumors once the diagnosis of LEMS is established. Almost all of SCLC are found within a year since LEMS diagnosis is made (71). Computed tomography (CT) of the thorax or 18F-fluorodeoxyglucose-positron emission tomography (PET), is recommended for oncological screening (72). Otherwise, paraneoplastic biomarkers may be useful for oncological screening as a supplementary to radiological investigations. Once diagnosis of tumor was made, it is of highest priority to treat the cancer at the same time. Surgical removal of cancers usually leads to a prominent alleviation of the symptoms, in which the underlying mechanism may probably because of the reducing VGCC antibodies and a reduction of the autoimmune response. If clinical remission is compromised and symptoms of LEMS remain, additional treatment, such as immunosuppressive treatment might bring symptomatic improvement.

#### Symptomatic Treatment

Symptomatic treatment of LEMS should aim to enhance the release of neurotransmitters from presynaptic nerve terminal or prolong the activity or availability of AChs in the synaptic cleft. The fundamental and effective symptomatic treatment of LEMS is 3,4-diaminopyridine (3,4-DAP), a drug that blocks VGKCs, prolongs nerve terminal depolarization and increases ACh release from nerve terminal (73, 74). Starting dose of 3,4-DAP is generally from 5 to 10 mg, 3–4 times per day. Most of the patients have a relatively good response from 40 to 60 mg/d. The suitable dose can gradually increase to 80 mg/d, divided into four to six times. Clinic improvement can always be detected within 30 min and reaches a peak at 90 min after each intake (75). 3,4-DAP is well-tolerated. Perioral tingling, digital paresthesias, and gastrointestinal symptoms are the most common side effects (76). Doses of more than 100 mg/d may increase the risk of seizures (77). Since QT interval prolongation was found in patient taking 3,4-DAP, thus before and during intaking 3,4-DAP, electrocardiogram (ECG) should be examined (74).

Theoretically, pyridostigmine, an AChE inhibitor used to prolong the AChs activity in the synapse cleft, is in synergy with 3,4-DAP, but many patients of LEMS seldom benefit from pyridostigmine either on its own or in combination with 3,4-DAP, which largely compromise the clinic use of pyridostigmine in LEMS (78, 79).

#### Immunosuppressive Agents

Since LEMS is caused by the antibodies against VGCCs, treatment suppressing the immune system is effective. If 3,4-DAP preferably manage the symptoms of LEMS, no further treatment is needed. If symptoms remain, long-term treatment of immunosuppressors, such as prednisone and azathioprine, should be considered, although the direct evidence for their efficacy in treating LEMS is somehow uncertain (80).

#### Other Treatment

Clinical guidelines of American Academy of Neurology (AAN) review the use of intravenous immunoglobulin (IVIg) in the treatment of neuromuscular disorders, including LEMS and MG (81). According to clinical studies, AAN has endorsed the clinical use of IVIg as supported by evidence of efficacy in the treatment of MG (level B) and LEMS (level C). Some reports and single randomized placebo-controlled crossover studies found clinical improvement in LEMS patients after treatment with IVIg, peaking at 2–4 weeks, and declining by 8 weeks (76, 82–84). Plasma exchange (PE) has been reported in case series and case reports but are lack of clinical trials in LEMS patients. Patients with LEMS respond more slowly to PE than do patients with MG, with a peak effect at almost 2 weeks, and the duration of effect may vary from 1 to 6 weeks (85). PE may result in short-term improvement of LEMS, but is not particularly effective in the management of LEMS without immunosuppressive medications and the other pharmaceutical approaches already mentioned (80, 86).

## Isaacs' Syndrome

Isaacs' syndrome is a rare autoimmune channelopathy at NMJ first characterized in 1961 by Hyam Isaacs in two patients with continuous muscle fiber activity (87). Since the patients were not ameliorated by peripheral nerve blockade but could benefit from curare, an inhibitor of AChRs, Isaacs later proposed that the reason of the spontaneous motor activity was due to the distal segments of peripheral nerves. Currently, Isaacs' syndrome is one of the most well-known peripheral nerve hyperexcitability (PNH) which causes persistent muscle fiber contraction characterized by muscle stiffness at rest and impaired muscle relaxation after voluntary contraction, yet different from myotonia (88). Clinically, Isaacs' syndrome is deemed as an autoimmune neuromyotonia disorder.

### Clinical Features

Isaacs' syndrome is a channelopathy with heterogeneity which affects patients at any age and varies significantly in severity. Little epidemiological data could be reviewed in previous literatures due to its rare occurrence and potential underestimation. Although Isaacs' syndrome is an autoimmune channelopathy, intriguingly, it was reported that male is more susceptible than female by ~2-folds. The average onset age is in the mid-40s (89–91).

Interestingly, several decades ago, Isaacs' syndrome was defined as “cramp-fasciculation syndrome” because of the chief complaint of cramps and fasciculation (92). About one third of patients have slow muscle relaxation after voluntary contraction, such as handgrip, eye and jaw closure, which is termed as pseudomyotonia (93, 94). In most cases, it manifests with muscle stiffness and muscle cramps worsen by voluntary muscle movement, which commonly without muscle weakness and muscle atrophy at beginning. On physical examination, marked fasciculation and myokymia can be noticed. Fasciculations are spontaneous discharges of a single motor axon which cause focal or multifocal single twitches in a group of muscle fibers, while on the other hand, myokymia are a numerous involuntary, undulating muscle twitches in wavelike style which are visible on the muscle surface. Visible myokymia is one of the most characteristic symptoms in Isaacs' syndrome, almost observed in 90% of patients (94). Even when myokymia is not visible, it is often palpable by clinician. Generally, it can be observed in the limbs, but also can be detected in other muscles, such as truncal and facial muscles (89, 95). Muscle cramps are also one of the frequent signs observed in Isaacs' syndrome in more than 70% of cases and usually can be painful (94). Muscle stiffness can be associated with cramps, which can also be present in rest or sleep and may improve after repeated exercise (96).

Other clinical manifestations include muscle hypertrophy which most often occurs in but not limit to calf muscles (97), sensory disorders which often manifest as distal hypesthesia in a small number of patients (89), and autonomic dysfunction, such as hyperhidrosis, sialorrhea, palpitations, flush, and abdominal pain (87).

### Pathophysiology

The fundamental pathophysiology of Isaacs' syndrome is dysfunction of VGKCs in presynaptic terminals due to acquired causes (98). Normally, Isaacs' syndrome is deemed as an autoimmune channelopathy while those neuromyotonia caused by genetic factors were usually classified as genetic diseases.

#### Antibody

Isaacs' syndrome is an autoimmune channelopathy at the NMJ caused by a group of autoantibodies. Several antibodies have been reported. However, there are almost 40% of patients have no defined targets (89, 99). Some VGKCs antibodies were detected in Isaacs' syndrome, however, positivity of antibodies against VGKCs in the absence of antibodies to leucine-rich glioma inactivated 1 (LGI1) and contactin-associated protein-like 2 (CASPR2) is not a clear disease biomarker for autoimmune inflammation and seems not to contribute in clinical practice (100). Antibodies against LGI1 and CASPR2 are antibodies against VGKCs-associated proteins rather than directly against VGKCs subunits, which were identified in 2010 (101). These antibodies do not directly block VGKCs, but rather decrease channel density either through increased degradation or decreased expression of VGKCs (102).

LGI1 is a secreted neuronal protein mainly expressed in the hippocampus specifically associated with VGKCs subunits in central nervous system presynaptic terminals (103). CASPR2 is a transmembrane protein expressed both in the central and peripheral nervous system with a large extracellular sequence which is vital for localization of subunits of VGKCs at juxtaparanodes (104). Not only detected in Isaacs' syndrome, both LGI1 and CASPR2 antibodies can also be discovered in other diseases, such as Morvan's syndrome, neuropathic pain, epilepsy, limbic encephalitis, and cerebellar dysfunction (105). LGI1 antibody seems to be more strongly associated with limbic encephalitis than Isaacs' syndrome and less seropositive in Isaacs' syndrome compared with CASPR2 antibody (106). It has been reported that these two antibodies highly correlate with clinical measures and have little correlation with cancers in Isaacs' syndrome (107). Since VGKC antibodies have little specificity in Isaacs' syndrome, the titers of antibodies should be considered cautiously during the clinical evaluation especially for those low positive tilters.

#### Paraneoplastic Association

Since male is more susceptible than female to Isaacs' syndrome by almost 2-folds, suggesting that paraneoplastic syndrome may be a cause of Isaacs' syndrome. More and more researches report that malignancies are found in patients with Isaacs' syndrome, supporting the hypothesis that tumor antigens trigger an autoimmune response and result in antibodies against VGKCs (94). The possible pathogenesis of paraneoplastic Isaacs' syndrome may be the activation of immune response by tumor-related antigens leading to autoantibodies, such as those targeting components of the VGKC complex (108). Thymoma and SCLC are the tumors most commonly associated with Isaacs' syndrome (109, 110).

## EMG

EMG shows characteristic myokymic and neuromyotonic discharges (111, 112). Sensory and motor nerve conduction studies are seldomly abnormal, including late responses, such as F waves and H reflexes, except for after discharges on motor nerve conduction studies. Myokymic discharges are spontaneous, continuous, rhythmic, irregularly occurring doublet, triplet or multiplet single motor unit discharges, with a frequency of around 30–40 Hz, followed by a short interval of silence, always up to a few seconds, and then recurrence of the burst at regular intervals (113). On the contrary, neuromyotonic discharges are composed of firing of single myofibers at high frequencies of 150–300 Hz. They can be spontaneous or be provoked by needle movement or muscle contraction. Repetitive supramaximal stimulation of a peripheral nerve at 10 Hz shows a sensitivity of 79% and specificity of 88% for identifying patients with Isaacs' syndrome. No direct evidence shows that SFEMG helps for detection of Isaacs' syndrome, thus SFEMG needs not be performed unless concerning exists for a defect of neuromuscular transmission, such as MG and LEMS (114).

### Treatment

Screening of tumor should be positively performed, especially thymoma (115), SCLC (109), and hematological tumor (116). Once tumors were detected, it is better to remove the cancer if it is possible. If no underlying tumors are detected, initial treatment should better include only symptomatic treatment.

#### Symptomatic Treatment

Currently, there are no FDA-approved drugs for symptomatic treatment of Isaacs' syndrome. Anticonvulsants are often used to moderate the symptoms of Isaacs' syndrome, such as cramps. Carbamazepine and phenytoin, which mainly work through sodium channel blockage, have been shown to be effective for Isaacs' syndrome (117). Gabapentin at a dose of up to 900 mg/day also appears to be beneficial, by predominantly affecting the central pain pathways through binding to calcium channel subunits (118–120). Carbamazepine is recommended as a first-line agent for symptomatic therapy at 400–600 mg/day in divided doses initially, with up to 1,200 mg/day in divided doses as tolerated (3). Efficacy of therapy should be assessed by monitoring the clinical response, rather than electrodiagnosis which can only be used as a secondary outcome measure.

#### Other Treatment

Beneficial effect of PE have been shown in many studies (121). PE can also be used in combination with prednisolone and azathioprine (122). PE is recommended as the first-line immunomodulating treatment for Isaacs' syndrome (3). IVIg, another common treatment for autoimmune disorders, has been reported to be less effective for Isaacs' syndrome (122).

## MG

MG is the most common autoimmune neuromuscular junction channelopathy caused by pathogenic autoantibodies to components, mostly are AChR, MuSK, and LRP4 on the post-synaptic muscle membrane (123). Patients usually complain about muscle weakness with fluctuations in severity in 1 day, which is a remarkable feature of MG. Increased muscle weakness after continued muscle activity represents a strong diagnostic clue for diagnosis of MG. The course of the disease is highly variable, symptoms and signs may change rapidly due to infection or pregnancy. Respiratory muscles may be involved leading to respiratory failure. Diagnosis should be based on confirmatory diagnostic testing, including serum antibodies tests and EMG. Treatment for MG traditionally contains thymectomy, AChE inhibitors, immunosuppressors, PE, and IVIg.

### Epidemiology

MG is the most common autoimmune NMJ channelopathy with a worldwide prevalence of 40–180 and an annual incidence of 4–12 per million people (124). AChR seropositive MG has an obvious age pattern of incidence, with a peak age of third decade which is a strong female predominance, and another peak in the elderly with a slight male predominance (125, 126). The incidence peak in young adults is partly due to of the high frequency in female which is typical for many autoimmune disorders, while late-onset MG is slightly more frequent in male (124, 127). The incidence of MuSK-associated MG in Netherland is estimated at 0.1 patients per million per year, with a prevalence of 1.9 per million people (128). In contrast to AChR seropositive MG, where the peak incidence is the third decade, age at symptom onset of MuSK-associated MG is distributed around a peak in the fourth decade, with another smaller peak in the second decade (129). MG rarely coincides in members of the same family (130, 131).

### Clinical Feature

Muscle weakness is the most common symptom in MG. Combination of fluctuation in muscle weakness over time and exercise-induced muscle weakness strongly implies the diagnosis of MG.

Muscle weakness in MG can occur in all the skeletal muscles including extraocular, bulbar, limb, and axial muscles. Over half of patients have prominent ptosis or diplopia, and in 20% patients, the muscle weakness is restricted in extraocular muscle without any other muscle weakness (132). Interestingly, weakness of extraocular muscles tends to be asymmetrical, while limb weakness is mostly symmetrical and more severe in proximal than distal (133). Ocular MG (oMG) is a more common form of juvenile MG in Asian populations than in other populations (134, 135). Patients may have eyelid retraction, most prominent upon awakening. If respiratory muscle weakness occurs, patients may develop respiratory failure requiring intubation (136). Premonitory signs usually include difficulty breathing, swallowing, and chocking. Speech can also be affected leading to a change in voice characteristics. Severity of MG can be quantified according to the Myasthenia Gravis Foundation of America's classification system (137).

Since MG is caused by autoantibodies, there is an increased frequency of organ-specific and general autoimmune disorders especially thyroiditis (138). Sixty-five percentage of MG patients have thymic hyperplasia and 10–15%, a thymoma. It is reported that the initial steps triggering humoral immunity in MG take place inside the thymic tissue and thymoma (139).

### Pathophysiology

Nowadays, MG is considered as a T-cell-mediated disease. The thymic tissue is able to express epitopes cross-reactive with skeletal muscle proteins, such as AChR, titin, and ryanodine receptor (RyR) (140). Thymic epithelial cells present AChR peptides to T cells in MG patients, resulting in intrathymic immunization (141). The immune response against epitopes expressed on abnormal thymic cells spills over to components at NMJ, mostly like AChR, which causes symptoms of MG (142).

#### Antibodies

MG is mainly caused by antibodies against AChR or other proteins on the post-synaptic membrane, with a characteristic of impaired signal transduction, muscle weakness, and fatigability. AChR antibodies are found in 85% of all MG patients (143). IgG1 and IgG3 are the prevalent subclass of AChR antibodies which have ability to activate complement and therefore to cause post-synaptic membrane damage and block the signaling pathway (144). Antibodies against AChR α subunit are more pathogenic than those against other subunits, such as β, δ, γ, and ε. Different AChR epitope antibody pattern influences disease severity (145).

MuSK is an AChR related membrane protein which is critical for the formation of NMJ (146). MuSK antibodies occur in < 10% of MG patients. In most MuSK-associated MG patients, MuSK antibodies are predominantly against the IgG4 subclass, a minor IgG component without well-defined, but presumably anti-inflammatory roles in immunity. Although IgG4 is deemed to have no activation effect on potent complement, MuSK antibodies bind to the extracellular N-terminal Ig-like domains of the AChR, retaining direct pathogenic capability by reducing post-synaptic AChR density, impairing the alignment between motor nerve terminal and post-synaptic membrane (147).

The prevalence of LRP4 antibodies represents in < 50% of AChR and MuSK antibodies double negative patients (148). In LRP4 immunized mice, LRP4 antibodies induce muscular weakness through disruption of the interaction between LRP4 and agrin, and thereby inhibit AChR-mediated neuromuscular signal transmission (149). Although the presence of anti-LRP4 in MG has been confirmed, their exact prevalence, pathogenic role and associated clinical phenotypes are largely unknown.

Neuronal agrin is an indispensable factor for formation of the NMJ by binding to LRP4 and stimulating MuSK (150). Agrin autoantibodies were detected in some MG patients, either with or without AChR or MuSK antibodies (151, 152). Agrin antibodies can inhibit MuSK phosphorylation and AChR clustering, which is detected in MG patients only (153).

### Clinical Classification

According to the age of onset, autoantibodies and thymic pathology, the disease forms are generally divided into several subgroups.

#### Pure oMG

In this form, muscle weakness is restricted to ocular muscles. Although this type is at risk of progressing to generalized MG (gMG), 90% of those who have had the ocular form for more than 2 years will remain in this subgroup (154). MuSK antibodies very rarely occur in this type (154).

#### Thymoma-Associated MG

10–15% of all patients associate with thymoma. Thymoma-associated MG is widely deemed as a paraneoplastic disease. Nearly all patients of this type are AChR-associated MG and seldom are ocular MG. Thymoma-associated MG patients usually have a higher prevalence of severe phenotype and also higher anti-AChR antibody titer than non-thymomatous MG patients (155).

#### AChR-Associated gMG

Nearly 85% of the MG population have detectable AChR antibodies and display this form of the disease. The titer of antibodies has no clear correlation with severity of the disease (156). Thymic abnormalities are more frequently found in this form than other types of MG (157). This form can be further categorized into two types: early onset MG that the onset of the disease before the age of 50, and late onset MG that the onset of the disease after the age of 50.

#### MuSK-Associated MG

Typically, MuSK antibody positive patients are female predominance, and they have a relatively severe form of the disease with muscular atrophy. The facial, bulbar, and respiratory muscles are frequently affected, while ocular muscle weakness and thymic abnormalities are rare (158, 159).

#### LRP4-Associated MG

LRP4 antibodies were discovered in ~12–50% of patients who were double seronegative for AChR and MuSK (160). The clinical phenotype of this type is not well-defined.

#### Antibody-Negative MG

MG patients lack of antibodies of AChR, MuSK, and LRP4 are traditionally called antibody-negative MG or seronegative MG. MG of this type represents a heterogeneous group pathogenically. Patients of this type probably have undefined pathogenic antibodies against proteins in the post-synaptic membrane (161). The diagnosis is more challenging in patients in whom no specific autoantibodies are detected.

### Diagnosis

Patients with classical fatigable symptoms need further examination. Ancillary tests include pharmacologic, serologic, and electrophysiologic tests.

#### Neostigmine Test

Intramuscular injection of 1.0–2.0 mg neostigmine, an AChE inhibitor, has a remarkable ameliorative effect on the deficit signs, such as ptosis, hypernasal voice, and limb weakness from 30 min and persisting for almost 90 min after injection (162). By inhibiting AChE through neostigmine, the amount of AChs is significantly increased in the synaptic cleft, and AChs are capable of binding to the AChRs for a longer period, resulting improved neuromuscular transmission. In MG, 90% patients response positively to AChE inhibitors. A positive reaction to AChE inhibitors can also be observed in congenital myasthenic syndrome, LEMS, amyotrophic lateral sclerosis and Guillain-Barrè syndrome (163). Nevertheless, although neostigmine test is much less used in the past, considering its easy methodology and inexpensive cost, it can still be recommended in developing countries. Although neostigmine test may be one of the first screening of MG, the responsiveness is not necessarily diagnostic for MG.

#### Serologic Test

The AChR antibodies are highly specific for MG diagnosis (123). If they are negative, it is important to test the anti-MuSK, LRP4 or other clustered AChR antibodies. Their presence is important as it can largely help to make the diagnosis of MG in some uncertain cases.

#### EMG

Strictly speaking, any AChE inhibitors should be stopped at least 12 h before EMG examination. The examination of EMG is pivotal for MG diagnosis and must be investigated in several proximal and distal nerve and muscle pairs. The classic electrophysiologic demonstration of an NMJ transmission defect is the documentation of a decremental response of the CMAP to slow (2–3 Hz) motor repetitive nerve stimulation (164). In RNS, a gold standard for MG, a decremental response of 10% from the first to the fourth or fifth response while stimulating at 2–5 Hz is valid for the diagnosis of MG.

SFEMG is a highly selective recording technique in which a concentric needle electrode is used to identify and record extracellular action potentials from individual muscle fibers (165). The typical SFEMG finding in MG is that increased jitters with impulse blocking, increased jitter without impulse blocking and also normal jitter can be detected within one muscle. Since SFEMG demonstrates abnormal jitter in virtually all patients with MG, it has been known to be the most sensitive diagnostic procedure for the diagnosis of MG for many years (166–168). Although SFEMG that reveals an elongated jitter is more sensitive than the RNS, it is not specific for MG, for example, in the radiculopathies and neuropathies, the specificity of SFEMG has been questioned (169–171). Besides the diagnostic value for MG, SFEMG is a valuable prognostic factor. In most MG patients, the changes in SFEMG measurements, especially the percentage of abnormal jitter pairs with blocking, correlated with the changes in clinical state as measured by quantitative testing of muscle function (172, 173).

#### CT Scan

Since a majority of MG patients have thymic diseases, it is essential to take a CT scan especially for gMG patients and those with anti-AChR antibodies (174). It is justifiable to control the thymus every 5 years if the patient was not thymectomized.

### Treatment

Therapies for MG include pharmacotherapy, such as symptomatic drugs, immunosuppressors, and other therapies, such as thymectomy, PE, and IVIg.

#### Symptomatic Treatment

Pyridostigmine, an AChE inhibitor, is the main pharmacologic compound used for MG, both in children and adults. If appropriate usage and dosage of pyridostigmine are prescribed, symptoms and signs of MG still have remission, other immunosuppressive treatment should better to use at the same time (136).

#### Immunosuppressive Agents

The most common immunosuppressive drug for MG is prednisone which has a good therapeutic effect generally. This medication can easily be administered orally even to children. Additionally, treatment with prednisone can protect the conversion from oMG to gMG (175, 176). Azathioprine can be considered as a second-line treatment for MG patients who respond poorly to prednisone treatment (177). Other immunomodulatory medications can be considered for use in MG, such as rituximab, mycophenolate mofetil, tacrolimus, and eculizumab, which are shown effective therapeutic efficacy and considered as second-line treatment combinated with or without prednisone in some clinic studies (178–182). It is worth mentioning that eculizumab, one of the latest generation treatment, was approved for the treatment of adults with AChR-associated gMG in the USA (183), AChR-associated refractory gMG in the EU (184) or patients with AChR-associated gMG whose symptoms are difficult to control with high-dose IVIg therapy or PE in Japan (185). One of a latest meta-analysis found that eculizumab is the most effective and tolerable therapeutic for refractory MG and tacrolimus is a beneficial therapy for MG extensively (186). Moreover, some new drugs are also under exploration which need further researches, such as efgartigimod (187).

#### Thymectomy

Many studies have reported a beneficial effect of thymectomy on MG (138). The thymus may trigger autoimmunity against AChRs, thus, its removal may eliminate the main source of antibody production against AChRs which alleviates symptoms of MG. For early-onset MG, thymectomy is recommended for MG, while in late-onset MG, thymectomy is debated (188). The latest researches support that thymectomy improves clinical outcomes even in patients with non-thymomatous gMG (189, 190). Thymectomy is also proved to be safe for juvenile MG, even down to an age of about 5 years (191). All thymus tissue needs to be removed. Since no direct therapeutic effect has been found for patients with MuSK, LRP4, and oMG, thymectomy is not recommended for these patients.

#### Other Treatment

IVIg and PE are two specific immunosuppressive treatments with a rapid and definite effect occurring often after 2–5 days, and either one often be given to patients with severe MG or MG crisis. IVIg can be administered for MG in an effort to reduce the circulating autoantibodies by decreasing B-cell antibody production and T-cell function. PE works primarily by removing circulating autoantibodies responsible for neuromuscular junction dysfunction, and also removing cytokines responsible for activating lymphocytes, irrespective of antibody status (123). Although IVIg and PE shows a comparable efficacy and duration of effect in MG patients, IVIg is often slightly more convenient, with a lower risk of severe side-effects, and less economic cost, whereas PE might work slightly more rapidly (192, 193). Elderly and those with complex comorbid diseases including acute respiratory failure may be better treated with IVIg (192).

## Differential Diagnosis

The autoimmune channelopathies at NMJ, LEMS, Isaacs' syndrome and MG, have overlapped clinical symptoms with each other, which renders the diagnosis more complicated. Auxiliary examinations are necessary for differential diagnosis. Key points of differential diagnosis are concluded in Table 1.

**Table 1:** Summary of main features of autoimmune neuromuscular junction channelopathies.

**Disorder**	**Epidemiology**	**Clinical manifestation**	**Autoantibody**	**Tumor**	**Electromyography**	**Treatment**
LEMS	2–4 per million, and 46 times less than that of MG. Female predominance before 45 years old, male predominance after 60 years old.	Proximal muscle weakness, autonomic features, areflexia.	anti-VGCC, anti-SOX1	SCLC	1. Massive decrease of CAMP. 2. Increase of the CMAP amplitude more than 100% in high-frequency stimulation (50 Hz).	1. Tumor treatment. 2. Symptomatic treatment: 3,4-DAP. 3. Immunosuppressors, prednisone, azathioprine. 4. PE and IVIg.
Isaacs' syndrome	Average onset age in the mid-40s. Female predominance.	Cramps after voluntary muscle movement, fasciculation.	anti-LGI1, anti-CASPR2	Thymoma, SCLC, hematological tumor	1. Intraburst spike frequency y30–40 Hz. 2. Single myofibers firing at 150–300 Hz, decrementing amplitude and waning firing pattern.	1. Tumor treatment. 2. Symptomatic treatment: carbamazepine. 3. PE and IVIg.
MG	40–180 per million. The most common autoimmune neuromuscular junction channelopathy. Female predominance.	Fluctant muscle weakness, exercise-induced muscle weakness.	anti-AChR, anti-MuSK, anti-LRP4	Thymic hyperplasia, thymoma	1. Decremental response of 10% in repetitive nerve stimulation. 2. SFEMG is sensitive for MG diagnosis.	1. Thymectomy. 2. Symptomatic treatment: pyridostigmine. 3. Immunosuppressors, prednisone, azathioprine. 4. PE and IVIg.

### Clinical Features

Among these channelopathies, Isaacs' syndrome is the most easily to differentiate from LEMS and MG. Isaacs' syndrome have prominent symptoms, such as cramp, fasciculation, and myokymia which are rarely detected in LEMS and MG. To differentiate LEMS and MG, the former one typically starts with mild leg weakness, which progresses in a caudocranial direction, while the latter one commonly begins with oculobulbar weakness, and muscle weakness spreads craniocaudally (194). Autonomic dysfunction and diminished tendon reflexes are rarely seen with MG, while are quite normal in LEMS (194). Once autonomic dysfunctions present in one patient, the diagnosis of MG should be doubtful.

### Auxiliary Examination

#### Electromyography

Needle electrode examination of Isaacs' syndrome normally shows an abnormal pattern of motor unit firing which are different with LEMS and MG, consisting of myokymic discharges, doublets and multiplets, neuromyotonic discharges, and fasciculations, which may occur spontaneously or may be activated by voluntary muscle contraction. These abnormalities may occur alone or in combination (195). To differentiate LEMS and MG, RNS is an indispensable test. Traditionally, an increase of the CMAP amplitude more than 100% is considered specific for LEMS. Recently, an increase of the CMAP amplitude more than 60% is considered to have both high sensitivity and specificity for LEMS diagnosis, which is rarely represented in MG (196).

#### Serological Test

Since all these disorders are autoimmune diseases, serological tests of antibody are necessary for diagnosis and identify the subgroup of disease. More than 85% of LEMS have antibodies against P/Q-type VGCC which are highly specific (55–57). While to MG, antibodies against AChR, MuSK, or LRP4, which also have high specificity for MG, are presented in almost more than 90% patients (123). For Isaacs' syndrome, antibodies have less sensitivity and specificity for diagnosis.

## Conclusion

At NMJ, three channels, VGCCs, VGKCs, and AChRs, play fundamental roles in signal transmission. Autoimmune antibodies of certain channels cause distinct symptoms. All the autoimmune neuromuscular junction channelopathies are orphan diseases causing by antibodies against these channels. Different groups of antibodies cause different disorders and symptoms. Although they share some common symptoms, characteristic symptoms or signs can be detected in different channelopathies, such as autonomic dysfunction, myokymia, fasciculation, and fluctuation of muscle weakness. Making a correct diagnosis of these channelopathies may be somehow hard, but when suspicion of neuromuscular junction channelopathies is raised for a fatigable deficit, proper diagnostic tests should be pursued. Besides the typical symptoms and signs, auxiliary examinations including EMG and serologic tests are essential for diagnosis. Since a part of patients with these disorders can associate with tumors, oncological screening tests should not be neglected. After diagnosis, appropriate treatment is pivotal for patients' quality of life and ability to perform daily activities. Due to current limited knowledge of these channelopathies, there is a need for a standard regimen for diagnosis and treatment of autoimmune neuromuscular junction channelopathies to maximize long-term benefits.

## Author Contributions

KH and HY conceived and planned the review. KH wrote the manuscript. Y-BL and HY critically revised the manuscript for important intellectual content.

### Conflict of Interest Statement

The authors declare that the research was conducted in the absence of any commercial or financial relationships that could be construed as a potential conflict of interest.
